# Characteristics and outcome of pregnancy-related acute kidney injury in a teaching hospital in a low-resource setting: a five-year retrospective review

**DOI:** 10.1186/s12882-024-03616-9

**Published:** 2024-05-22

**Authors:** Ephrem Berhe, Hale Teka, Hiluf Ebuy Abraha, Bisrat Tesfay Abera, Marta Abrha Gebru, Tsega Gebremariam, Mohammedtahir Yahya, Birhane Amare, Habtom Tadesse, Hagos Gidey, Fireweyni Tesfay, Mohamedawel Mohamedniguss Ebrahim, Rahel Kidanemariam, Awol Yemane Legesse

**Affiliations:** 1 Department of Internal Medicine, Nephrology Unit, School of Medicine, Mekelle University, Mekelle, Tigray Ethiopia; 2 Department of Obstetrics and Gynecology, School of Medicine, Mekelle University, Mekelle, Tigray Ethiopia; 3 Department of Biostatistics, School of Public Health, Mekelle University, Mekelle, Tigray Ethiopia; 4 Department of Epidemiology and Biostatistics, University of South Carolina, Arnold School of Public Health, PO. Box: 1871, Columbia, SC USA

**Keywords:** Pregnancy-related acute kidney injury, Outcome, Determinant factors, Resource-limited setting, Ethiopia

## Abstract

**Background:**

Pregnancy-related kidney injury contributes to a high burden of acute kidney injury in low-resource settings and causes maternal and perinatal morbidity and mortality. Few studies have examined the impact of acute kidney injury in resource-limited countries, with very limited research on pregnancy-specific disorders in Ethiopia. This study aimed to determine the characteristics of pregnancy-related acute kidney injury, outcomes and associated factors.

**Methods:**

A retrospective study was conducted to evaluate the clinical profile and maternal-fetal outcome of pregnancy-related acute kidney injury at Ayder Comprehensive Specialized Hospital in Tigray, Ethiopia, from January 1, 2017, to December 31, 2021. Maternal and fetal outcomes were analyzed using descriptive statistics. Multivariate logistic regression was used to determine the association between the dependent and independent variables.

**Results:**

Of 27,350 mothers who delivered at Ayder Comprehensive Specialized Hospital between January 1, 2017, and December 31, 2021, a total of 187 women developed pregnancy-related acute kidney injury, a prevalence rate of 68 per 100,000 births. Preeclampsia, sepsis and pre-renal causes due to dehydration and hemorrhage were the most common causes of pregnancy-related acute kidney injury in this study. Hemodialysis was needed in 8.6% (n = 16) of patients. Of the 187 pregnancy-related acute kidney injuries, 143 (76.5%) recovered completely and 30 (16%) partially. The mortality rate was 7.5%. Preexisting chronic kidney disease (AOR = 30.13; 95% CI: 2.92, 310.84), use of vasoactive agents (AOR = 5.77; 95% CI: 1.47, 22.67), increase in creatinine per unit (AOR = 1.65; 95% CI: 1.11, 2.45) and complications related to acute kidney injury (AOR = 5.26; 95% CI: 1.73, 16.00) were determinants of the composite endpoints (partial renal recovery and death).

**Conclusions:**

This study emphasizes acute kidney injury in resource-limited settings is a significant cause of maternal and fetal morbidity and mortality. The vast majority of patients with pregnancy-related acute kidney injury recovered completely from kidney injury. The main causes of pregnancy-related acute kidney injury were preeclampsia, sepsis and pre-renal associated with hemorrhage and dehydration. Preexisting renal disease, use of vasopressors, increase in creatinine per unit and complications associated with acute kidney injury were determining factors for concomitant fetomaternal mortality. Appropriate preventive strategies during prenatal care and prompt treatment are needed for pregnancy-related acute kidney injury.

## Introduction

Pregnancy-related acute kidney injury (PR-AKI) is a heterogeneous disorder that encompasses all causes of acute renal impairment from early pregnancy to 3 months postpartum [1]. It is an important obstetric complication associated with significant maternal and fetal morbidity and mortality [2]. Although the incidence of PR-AKI has decreased significantly in developed countries due to improved general health care, it remains a serious public health problem in developing countries [3]. A recent meta-analysis found an overall prevalence rate of 2% [4], and 3–5% of all AKI cases in developing countries such as India are due to PR-AKI [5]. Acute kidney injury (AKI) in sub-Saharan Africa is a major challenge due to the late presentation of patients to health facilities and the lack of resources to care for patients with established AKI [6]. In Africa, PR-AKI is the second most common cause of AKI [7]. In Ethiopia, among patients with AKI requiring dialysis, 18.5% of AKI is attributed to pregnancy-related causes [8].

The etiology and presentation of AKI differ between high-income and low-income countries [9]. In Africa, the most commonly reported causes are infections, nephrotoxins, and obstetric and surgical complications [10]. The main conditions associated with AKI in the antenatal and postpartum period are preeclampsia/eclampsia, obstetric hemorrhage, HELLP syndrome and postpartum sepsis [11]. During the first trimester of pregnancy, AKI is commonly reported, secondary to pre-renal AKI due to dehydration from hyper emesis gravidarum and septic shock associated with sepsis [12]. In the third trimester and immediate Puerperium, it is associated with preeclampsia/eclampsia, ante partum hemorrhage (APH), postpartum hemorrhage (PPH), puerperal sepsis, hemolytic uremic syndrome (HUS) and HELLP syndrome [13].

Regarding the outcome of this disease, complete renal recovery is usually achieved if these patients are treated appropriately and promptly. However, pregnancy-related AKI has high impact on morbidity and mortality in both maternal and fetal outcomes. Inadequate initial resuscitation, late initiation of antibiotics, prolonged intervals until an appropriate clinical setting is reached and consequently significantly delayed initiation of dialysis are the main reasons for poor outcomes [14]. The maternal mortality rate (MMR) increases with increasing severity of pregnancy-related complications and can range from 13 to 24% in developing countries due to PR-AKI [15]. In Ethiopia, maternal mortality has changed significantly in recent decades. MMR decreased from 871 per 100,000 in 2000 to 401 per 100,000 in 2017, which corresponds to the death of approximately 12,000 mothers per year. Direct obstetric complications are responsible for 85% of these deaths [16]. In addition, fetal, neonatal and perinatal mortality is particularly high in newborns born to mothers with pregnancy-related AKI [17]. Therefore, pregnancy-related AKI leads to serious complications and higher maternal and fetal mortality [18]. Although low- and middle-income countries (LMICS) suffer the most from PR-AKI, few studies have investigated its prevalence and impact in obstetric patients. Therefore, this study sought to examine the prevalence, causes and outcomes of pregnancy-related AKI at Ayder Comprehensive Specialized Hospital, a tertiary medical center in northern Ethiopia.

## Methods

### Study design

A facility based retrospective study design was conducted at Ayder Comprehensive Specialized Hospital, Tigray, Ethiopia, to assess pregnancy-related AKI causes, outcomes and determine factors associated with severe maternal outcomes.

### Study period

The medical charts of all women who were diagnosed with pregnancy-related AKI between January 1, 2017, and December 31, 2021, were reviewed.

### Study setting

The study was carried out at ACSH, a tertiary care and teaching hospital located in the Tigray region of northern Ethiopia. It is the largest referral center in the Tigray region, with a catchment area of more than 8 million people from the Tigray region and neighboring districts of the Afar and Amhara regions. There are 500 inpatient beds. The hospital’s services include obstetrics and gynecology which is run by 20 consultants and 150 midwives. There are separate antenatal care clinics for low- and high-risk patients. The number of deliveries is increasing annually, and the hospital currently records an average of 5000 deliveries per year. Ayder Hospital’s hemodialysis center was established under a public-private partnership model. It is equipped with 12 modern B-Braun Dialog plus hemodialysis machines and is the only dialysis center in the region available for patients with acute kidney injury and chronic kidney disease, with the number of annual registrations for the dialysis unit increasing. The unit is staffed by 11 dialysis nurses and two adult nephrologists and has cared for almost 600 patients with kidney disease requiring dialysis since it was established in 2013.

### Study population

The records of all women who developed AKI and needed dialysis during pregnancy, childbirth, the post-abortion period and/or the Puerperium during the study period were included.

### Sample size and power

The charts of all women who were diagnosed with pregnancy-related AKI between January 1, 2017 and December 31, 2021 were reviewed. The list of mothers was compiled from the maternity delivery logbook, the operating theater logbook, the emergency logbook, and the ICU admission registers. Due to the rarity of pregnancy-related AKI, all cases were collected consecutively rather than employing a sampling technique. We computed the statistical power for a cohort comprising 187 mothers consecutively diagnosed with pregnancy-related AKI. Using the single population proportion formula, a significance level of α = 0.05, and a minimum effect size of 0.2, the calculated power was 0.87.

### Data collection tool and procedures

Initially, we identified AKI cases by cross-referencing medical record numbers with a separate logbook dedicated to near misses. Subsequently, we obtained and analyzed 187 AKI charts, using a questionnaire developed based on existing literature. A pilot test was then conducted to assess the tool’s usability and identify potential problems, leading to subsequent refinements. Open Data Kit (ODK) tool was utilized for data collection. Data extraction from patient records encompassed various aspects including, Sociodemographic characteristics, obstetric profile, Comorbidity, clinical presentation, investigation, management, and outcomes of women with pregnancy-related AKI. Trained medical doctors were responsible for collecting the data. Study participants were enrolled if they had [1] Absolute serum creatinine ≥ 1.2 mg/dl; [2] elevation of serum creatinine by 0.3 mg/dl within 48 h from their baseline; [3] decreased urine output for ≥ 6 h; and [4] the need for dialysis.

To guarantee the quality of the data, medical doctors involved in data collection underwent one-week training. Moreover, throughout the data collection process, investigators conducted daily supervision to ensure data completeness, accuracy, and validity.

### Measurement

#### AKI

AKI was defined according to the Kidney Disease Improving Global Outcomes (KDIGO) 2012 criteria as the increase in the absolute value of the SCr by 0.3 mg/dl within 48 h or an increase > 50% compared to the baseline values within 7 days, or a urine output < 0.5 mL/kg/h ≥ 6-hour [19].

#### Pregnancy-related AKI (PR-AKI)

all causes of acute renal function impairment from early pregnancy to three months postpartum [20].

#### Complete renal recovery

Serum creatinine at day 7 returned to baseline creatinine or to < 1.5x baseline [21] or returned to within 50% above baseline serum creatinine [22].

#### Partial renal recovery

No need for renal replacement therapy (RRT) but did not meet the criteria for complete renal recovery or creatinine persistently ≥ 1.5 mg/dL at day 7.

#### CKD

abnormalities of kidney structure or functional abnormalities with GFR ≤ 60 mL/min/1.73 m^2^ that is present for more than three months [23].

#### Composite endpoint

Creatinine persistently > 1.5 mg/dl indicating partial renal recovery at day 7 or death attributed to PR-AKI.

### Data analysis

We entered the data into the Open Data Kit Tool (ODK); the data were then transferred to the statistical software Stata version 16 and analyzed. Descriptive statistics are presented in the form of frequencies and percentages. After we checked the normal distribution of the data, the measures of central tendency and dispersion were reported using the mean with its standard deviation (SD) or the median with its interquartile range (IQR). The chi-square test or Fisher’s exact test was used to compare categorical variables, depending on the expected cell count. The difference between continuous variables was compared using an independent t-test.

Bivariate analysis was performed to examine the association between the independent variables and the composite endpoint outcome (creatinine > 1.5 mg/dl (partial renal recovery) or death). Variables that showed an association with the composite endpoint (with a p-value of < 0.25) were analyzed using multivariate logistic regression to assess whether there was a significant association between each independent variable. Independent variables that were associated with the outcome variable at a P value of < 0.05 were considered statistically significant. Multicolinearity diagnostics were performed, and collinear variables were excluded from the final model. The fit of the final model was tested using the Hosmer‒Lemeshow goodness-of-fit model. A receiver operating characteristic (ROC) analysis was also performed to estimate the predictive power of the final fitted model.

### Ethical clearance

This is a secondary analysis of data for a study on maternal near misses. Ethical approval was granted by the Institutional Review Board (IRB) of Mekelle University, University of Health Sciences, with ethical approval number MU-IRB 1950/2022. We were unable to obtain informed consent from the study participants, as the study was a retrospective design. However, a letter of support was obtained from the hospital’s medical director’s office, and patient profiles and data were granted full anonymity. The IRB also waived the requirement to obtain informed consent after reviewing the protocol for this study.

## Results

### Demographic and clinical characteristics

In the five years in which the hospital hosted 27,350 deliveries, 187 developed AKI. The median age of the participants was 27 years (IQR = 5) years. Most (51.3%) of the participants were rural dwellers. A total of 78 (41.7%) of the mothers were gravid one (range, 0–10). More than half (73.3%) had antenatal care follow-ups; 44 (23.5%) ended their pregnancy with spontaneous abortions. Regarding pregnancy outcomes, 100 (53.5%) mothers delivered vaginally. The median systolic and diastolic blood pressures at diagnosis were 140 (IQR = 40) and 90 (IQR = 30), respectively (Table 1).

**Table 1:** Demographic and clinical characteristics of the study participants, Ayder Comprehensive Specialized Hospital, Mekelle, Northern Ethiopia, 2017–2021 (n = 187)

Characteristic				Total (n = 187)	Composite endpoint		P-value
					Yes (n = 44)	No (n = 143)	
Age, [median (IQR)] (range, 17–50)				27 [5]	30 [10]	26 [7]	0.079
Age, n (%)							
	18–39			177 (94.6)	42 (95.5)	135 (94.4)	0.787
	*≥* 40			10 (5.4)	2 (4.5)	8 (5.6)	
Residence, n (%)							
	Urban			91 (48.7)	18 (40.9)	73 (51.0)	0.239
	Rural			96 (51.3)	26 (59.1)	70 (48.9)	
Region, n (%)							
	Tigray			155 (82.9)	37 (84.1)	118 (82.5)	0.257
	Afar			22 (11.8)	3 (6.8)	19 (13.3)	
	Amhara			10 (5.3)	4 (9.1)	6 (4.2)	
Gravidity, n (%)							
	Gravida 1			78 (41.7)	12 (27.3)	66 (46.1)	0.077
	Gravida 2–4			68 (36.4)	19 (43.2)	49 (34.3)	
	Gravida ≥ 5			41 (21.9)	13 (29.5)	28 (19.6)	
GA at admission [median (IQR)]				34 [2]	33 [3]	34 [2]	0.916
Trimester, n(%), n = 111							
			First	3 (2.70)	1 (6.67)	2 (2.08)	0.455
			Second	16 (14.41)	3 (20.00)	13 (13.54)	
			Third	92 (82.89)	11 (73.33)	81 (84.38)	
Antenatal care, n (%)				137 (73.3)	26 (59.1)	111 (77.6)	0.015
Spontaneous abortion, n (%)				44 (23.5)	13 (29.5)	31 (21.7)	0.282
Pregnancy outcome, n (%)							
	Vaginal delivery			100 (53.5)	31 (70.5)	69 (48.2)	0.022
	Caesarean delivery			70 (37.4)	8 (18.2)	62 (43.4)	
	Evacuation			15 (8.0)	4 (9.1)	11 (7.7)	
	Dead/discharged while pregnant			2 (1.1)	1 (2.3)	1 (0.7)	
SBP at diagnosis [median (IQR)]				140 [24]	135 (50.5)	140 [25]	0.018
DBPat diagnosis [median (IQR)]				90 [26]	83 [26]	90 [26]	0.020
PR at diagnosis (per minute), n (%)							
		≤ 100		104 (55.6)	22 (50.0)	82 (57.3)	0.391
		> 100		83 (44.4)	22 (50.0)	61 (42.7)	
Temperature at diagnosis, n (%)							
		≤ 37.7		155 (82.9)	38 (86.4)	117 (81.8)	0.484
		> 37.7		32 (17.1)	6 (13.6)	26 (18.2)	
Oxygen		Saturation [Median (IQR)]		92 [10]	90 [10]	94 [12]	0.855

IQR: interquartile range, GA: gestational age, SBP: systolic blood pressure, DBP: diastolic blood pressure, PR: pulse rate

### Causes and co morbidities of pregnancy-related acute kidney injury

Hypertensive disorders of pregnancy 141(75.4%), sepsis 92(49.2%) and pre renal AKI 76(40.6%) attributed to hemorrhage and dehydration are the major causes associated with pregnancy-related AKI. Co morbidities were present in 44 (23.5%) of those with PR-AKI and the predominant ones identified are chronic liver disease in 15 (8%), pre existing chronic kidney disease in 11 (5.9%) and heart disease in 9 (4.8%) (Table 2).

**Table 2:** Causes and co-morbidities of pregnancy related acute kidney injury (PR-AKI), Ayder Comprehensive Specialized Hospital, Mekelle, Northern Ethiopia, 2017–2021 (n = 187)

Characteristic				Total (n = 187)	Composite end point		P-value
					Yes (n = 44)	No (n = 143)	
Hypertensive disorders of pregnancy, n (%)				141 (75.4)	28 (63.6)	113 (79.0)	0.038
			Pre-eclampsia, n (%)	133 (71.1)	24 (54.6)	109 (76.2)	0.006
			Eclampsia, n (%)	42 (22.5)	12 (27.3)	30 (21.0)	0.382
			Gestational hypertension, n (%)	40 (21.4)	8 (18.2)	32 (22.4)	0.553
•			Superimposed preeclampsia, n (%)	8 (4.3)	1 (2.3)	7 (4.9)	0.452
Sepsis, n (%)				92 (49.2)	26 (59.1)	66 (46.1)	0.133
Focus of sepsis, n (%)							
	Puerperium			19 (20.6)	4 (15.4)	15 (22.8)	0.052
	Gastrointestinal			7 (7.6)	5 (19.2)	3 (3.0)	
	Chest/Pulmonary			26 (28.3)	10 (38.5)	16 (24.2)	
	Urinary tract			4 (4.3)	1 (3.8)	3 (4.6)	
	Central nervous system			2 (2.2)	0 (0.0)	2 (3.0)	
	Pelvic			34 (37.0)	6 (23.1)	28 (42.4)	
Pre-renal AKI, n (%)				76 (40.6)	22 (50.0)	54 (37.8)	0.148
HELLP syndrome, n (%)				50 (26.7)	15 (34.1)	35 (24.5)	0.208
HUS, n (%)				1 (0.5)	0 (0.0)	1 (0.7)	0.309
AFLP, n (%)				3 (1.6)	1 (2.3)	2 (1.4)	0.687
Acute glomerulonephritis, n (%)				3 (1.6)	3 (6.8)	0 (0.0)	0.002
Comorbidity, n (%)				44 (23.5)	16 (36.4)	28 (19.6)	0.022
			Heart disease, n (%)	9 (4.8)	1 (2.3)	8 (5.6)	0.368
			Diabetes mellitus, n (%)	4 (2.1)	2 (4.5)	2 (1.4)	0.207
			HIV, n (%)	2 (1.1)	1 (2.3)	1 (0.7)	0.375
			Chronic hypertension, n (%)	8 (4.3)	1 (2.3)	7 (4.9)	0.452
			Preexisting CKD, n (%)	11 (5.9)	10 (22.7)	1 (0.7)	< 0.001
			Chronic liver disease, n (%)	15 (8.0)	4 (9.1)	11 (7.7)	0.765
Medication exposure, n (%)				142 (75.9)	32 (72.7)	110 (76.9)	0.569
		Vancomycin		24 (16.9)	10 (31.2)	14 (12.7)	0.014
		NSAIDS		33 (23.2)	8 (25.0)	25 (22.7)	0.789
		ACE inhibitors		3 (2.1)	1 (3.1)	2 (1.8)	0.651
		Magnesium sulfate		117 (82.4)	24 (75.0)	93 (84.5)	0.212
		Radiographic contrast		3 (2.1)	1 (3.1)	2 (1.8)	0.651
		Tenofovir		2 (1.4)	1 (3.1)	1 (0.9)	0.349

### Laboratory findings of the pregnancy-related AKI patients

Creatinine levels above or equal to 1.2 were observed in almost all 180 patients (96.3%). The maximum creatinine level for those with composite endpoints was 3.8 mg/dl. Positive dipstick albuminuria was observed in 107(69.5%) patients. Of the 97 participants whose urine output was monitored, 78(80.4%) had non-oliguric AKI. One-third of the patients had leukocytosis, and half of the patients had anemia (Table 3).

**Table 3:** Laboratory findings of the study participants, Ayder Comprehensive Specialized Hospital, Mekelle, Northern Ethiopia, 2017–2021 (n = 187)

Characteristic		Total (n = 187)	Composite endpoint		P-value
			Yes (n = 44)	No (n = 143)	
White blood cells (/microL), n (%)					
	4,000–10,000	114 (60.9)	21 (47.7)	93 (65.0)	0.108
	< 4,000	8 (4.3	2 (4.5)	6 (4.2)	
	> 10,000	65 (34.8)	21 (47.7)	44 (30.8)	
Hemoglobin (g/dL), n (%)					
	<=11)	98 (52.4)	32 (72.7)	66 (46.1)	0.008
	11.1–15.9	72 (38.5)	9 (20.5)	63 (44.1)	
	>=16	17 (9.1)	3 (6.8)	14 (9.8)	
Platelet count (/microL), n (%)					
	< 50,000	38 (20.3)	12 (27.3)	26 (18.2)	0.354
	50,000–100,000	34 (18.2)	6 (13.6)	28 (19.6)	
	100,000–150,000	115 (61.5)	26 (59.1)	89 (62.2)	
ALT [median (IQR)]		65 (120)	78 (145.5)	65 (117)	0.055
AST [median (IQR)]		66 (120)	83.5 (146.5)	63 (119)	0.169
Creatinine at diagnosis (mg/dl), n (%)					
	< 1.5	81 (43.3)	8 (18.2)	73 (51.0)	< 0.001
	1.5–1.9	54 (28.9)	11 (25.0)	43 (30.1)	
	2.0-2.9	25 (13.4)	7 (15.9)	18 (12.6)	
	≥ 3.0	27 (14.4)	18 (40.9)	9 (6.3)	
Maximum creatinine [median (IQR)]		1.6 (1.1)	3.8 (5.0)	1.5 (0.7)	< 0.001
Creatinine at discharge [median (IQR)]		1.0 (0.6)	1.8 (2.4)	0.8 (0.5)	< 0.001
Urea [median (IQR)]		58 [27]	105 (85)	47 [28]	< 0.001
Direct bilirubin [median (IQR)]		0.3 [1]	1 (4.7)	0.2 (0.3)	0.054
Indirect bilirubin [median (IQR)]		0.4 (1.5)	0.7 (2.2)	0.3 (0.6)	0.902
Albumin [median (IQR)]		2.1 (1.6)	1.9 (1.1)	2.6 (2.9)	0.307
Potassium [mean (SD)], n = 123		123 (4.5)	5.0 (1.5)	4.2 (0.8)	< 0.001
Sodium [median (IQR)], n = 86		135 [11]	132 [16]	136 [6]	0.158
Urine albumin, n (%), n = 154					
	Positive	107 (69.5)	28 (68.3)	79 (69.9)	0.847
	Negative	47 (30.5)	13 (31.7)	34 (30.1)	
Urine output in 24 h (ml/hour), n (%)					
	< 100	4 (4.1)	3 (12.0)	1 (1.4)	< 0.001
	100–400	15 (15.5)	9 (36.0)	6 (8.3)	
	> 400	78 (80.4)	13 (52.0)	65 (90.3)	

### Process of care and outcomes of the pregnancy-related AKI patients

Nearly one-third of patients with pregnancy-related AKI were admitted to the intensive care unit, and almost half of the patients needed mechanical ventilation. Vasoactive agents and parenteral antibiotics were used in 26(14%) and 109(58.3%) patients, respectively. Hemodialysis was offered in 16 (8.6%) patients with obstetric AKI. Of those who had pregnancy-related AKI 165 (88.2%) of them had recovery of their serial serum creatinine values to less than 1.5times of their baseline at around day 7. One-third of the patients had AKI-related complications. Anemia, impaired consciousness and hypertension were the predominant complications noted in 45 (71.4%), 26 (41.3%) and 19 (30.2%) patients, respectively. Live birth occurred in more than half of the mothers, while stillbirth and IUFD occurred in 33(17.7%) and 20(10.7%) mothers, respectively (Table 4).

**Table 4:** Outcome of the study participants, Ayder Comprehensive Specialized Hospital, Mekelle, Northern Ethiopia, 2017–2021 (n = 187)

Characteristic			Total (n = 187)	Composite endpoint		P-value
				Yes (n = 44)	No (n = 143)	
ICU admission, n (%)			50 (26.7)	23 (52.3)	27 (18.9)	< 0.001
Duration of ICU stay [median (IQR)]			5 [5]	5 [6]	6 [8]	0.792
Use of mechanical ventilation, n (%)			24 (48.0)	12 (52.2)	12 (44.4)	0.586
Use of vasoactive, n (%)			26 (13.9)	13 (29.5)	13 (9.1)	0.001
Use of parenteral antibiotics, n (%)			109 (58.3)	34 (77.3)	75 (52.4)	0.003
Hemodialysis, n (%)			16 (8.6)	10 (22.7)	6 (4.2)	< 0.001
Length of hospital stay [median (IQR)]			8 [10]	10 (9.5)	8 [9]	0.529
KDIGO recovery stages and serum creatinine at day 7, n (%)						
• Stage 0		• Serum Cr < 1.5x baseline	165 (88.2)	35 (79.5)	130 (90.9)	0.041
• Stage 1		• Serum Cr 1.5-1.9x baseline	9 (4.8)	1 (2.3)	8 (5.6)	0.368
• Stage 2		• Serum Cr 2.0-2.9x baseline	3 (1.6)	3 (6.8)	0 (0.0)	0.002
• Stage 3		• Serum Cr ≥ 3.0x baseline	10 (5.35)	5 (11.4)	5 (3.5)	0.0043
AKI-related complications, n (%)			63 (33.7)	29 (65.9)	15 (34.1)	< 0.001
	Hypertension		19 (30.2)	10 (34.5)	9 (26.5)	0.490
	Pulmonary edema		12 (19.0)	8 (27.6)	4 (11.8)	0.111
	Anemia		45 (71.4)	21 (72.4)	24 (70.6)	0.873
	Hyperkalemia		20 (31.7)	14 (48.3)	6 (17.6)	0.009
	Metabolic acidosis		13 (20.6)	8 (27.6)	5 (14.7)	0.208
	Altered mentation		26 (41.3)	17 (58.6)	9 (26.5)	0.01
Fetal outcome, n (%)						
	Born alive		105 (56.1)	22 (50.0)	83 (58.0)	0.478
	Stillbirth		33 (17.7)	8 (18.2)	25 (17.5)	
	IUFD		20 (10.7)	5 (11.3)	15 (10.5)	
	ENND		3 (1.6)	0 (0.0)	3 (2.1)	
	Abortus		20 (10.7)	8 (18.2)	12 (8.4)	
	Unknown		6 (3.2)	1 (2.3)	5 (3.5)	
If alive						
1st minute APGAR score [mean (SD)]			7.1 (1.2)	7.1 (0.8)	7.1 (1.3)	0.894
5th minute APGAR score [mean (SD)]			8.3 (1.0)	8.2 (0.5)	8.3 (1.1)	0.602
Birthweight in gram [mean (SD)], n = 95			2379 (848)	2800 (733)	2300 (849)	0.035

ALT: AST: IQR: AKI: APGAR

### Factors associated with the composite endpoint (partial renal recovery and death)

A total of 14 (7.5%) patients with pregnancy-related AKI died, and 30 (16.0%) were discharged with a creatinine level above 1.5 mg/dl, yielding a total of 44 (23.5%) patients with a composite endpoint. Table 5 summarizes the factors that were associated with the composite endpoint. In the multivariable analysis, patients with preexisting CKD had a higher risk of developing the composite endpoint than their counterparts (AOR = 30.13; 95% CI: 2.92, 310.84). For every one-unit increase in creatinine, the odds of reaching the composite endpoint increased by 65% (AOR = 1.65; 95% CI: 1.11, 2.45). Similarly, patients taking vasoactive agents were six times more likely to either die or be discharged with a creatinine above 1.5 mg/dl (AOR = 5.77; 95% CI: 1.47, 22.67). Patients with AKI-related complications were also highly likely to reach the composite endpoint (AOR = 5.26; 95% CI: 1.73, 16.00) (Table 5).

**Table 5:** Factors associated with the composite endpoint, Ayder Comprehensive Specialized Hospital, Mekelle, Northern Ethiopia, 2017–2021 (n = 187)

Characteristic		COR (95% CI)	AOR (95% CI)	P-value
Age (per year)		1.04 (0.99, 1.10)	0.99 (0.90, 1.09)	0.839
Gravidity				
	Gravida 1	1	1	
	Gravida 2–4	2.13 (0.95, 4.80)	1.77 (0.59, 5.31)	0.305
	Gravida ≥ 5	2.55 (1.04, 6.28)	3.08 (0.63, 15.09)	0.164
Antenatal care		0.42 (0.20, 0.85)	1.09 (0.37, 3.20)	0.877
SBP at diagnosis		0.99 (0.98, 0.99)	0.97 (0.94, 1.01)	0.173
DBP at diagnosis		0.98 (0.97, 0.99)	1.03 (0.98, 1.09)	0.195
Prerenal		1.65 (0.83, 3.26)	0.94 (0.32, 2.74)	0.918
Sepsis		1.68 (0.85, 3.34)	0.39 (0.11, 1.38)	0.143
Comorbidity*		2.35 (1.12, 4.92)	2.08 (0.76, 5.70)	0.154
Preexisting CKD**		41.76 (5.18, 337.46)	30.13 (2.92, 310.84)	**0.004**
Hemoglobin				
	Normal (11.1–15.9)	1	1	
	Anemia ( < = 11)	3.39 (1.50, 7.68)	1.39 (0.47, 4.09)	0.546
	Polycythemia ( > = 16)	1.50 (0.36, 6.26)	1.21 (0.22, 6.61)	0.821
ALT (per one unit)		1.00 (0.99, 1.00)	1.00 (0.99, 1.00)	0.170
AST (per one unit)		1.00 (0.99, 1.00)	0.99 (0.99, 1.00)	0.745
Creatinine (increment per one unit)		1.75 (1.37, 2.25)	1.65 (1.11, 2.45)	**0.014**
Urea (increment per one unit)		1.01 (1.01, 1.02)	1.00 (0.99, 1.01)	0.399
ICU admission		4.70 (2.28, 9.71)	1.57 (0.48, 5.11)	0.453
Use of vasoactive		4.19 (1.77, 9.94)	5.77 (1.47, 22.67)	**0.012**
Use of parenteral antibiotics		3.09 (1.42, 6.71)	0.79 (0.20, 3.10)	0.734
Hemodialysis		6.71 (2.28, 19.77)	3.21 (0.54, 18.95)	0.199
AKI related complication		6.20 (2.98, 12.90)	5.26 (1.73, 16.00)	**0.003**

Hosmer-Lemeshow goodness: chi2 [8] = 4.65, P-value = 0.795. The area under ROC curve = 0.86

SBP: systolic blood pressure, DBP: diastolic blood pressure, COR: crude odds ratio, AOR: adjusted odds ratio

*Controlled for all but preexisting CKD

**Controlled for all but Comorbidity

**Fig. 1:**
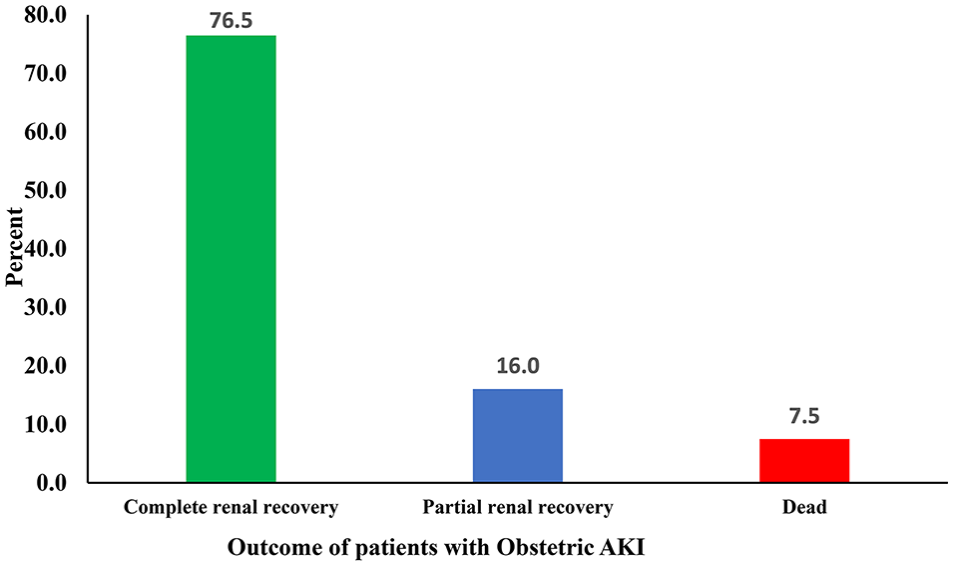
Maternal outcome among pregnancy related acute kidney injury patients. Ayder Comprehensive Specialized Hospital, Mekelle, Northern Ethiopia, 2017–2021 (n = 187)

As Fig. 1 clearly shows, of the women with PR-AKI (n = 187), 76.5% had achieved a complete renal recovery that was a cure from their AKI.

## Discussions

Among 27,350 parturient mothers hospitalized in the Department of Obstetrics in a tertiary hospital in northern Ethiopia, the incidence of AKI during pregnancy and puerperium was 0.68%. In the present study, the main causes of PR-AKI were hypertensive disorders of pregnancy, sepsis and pre renal AKI resulting from APH/PPH and dehydration. In more than two-thirds of the patients with PR-AKI, the kidneys recovered completely. Sixteen women (8.6%) received renal replacement therapy. The mortality rate among women with pregnancy-related AKI due to renal injury and its complications was 7.5%. The presence of preexisting CKD, increment of creatinine per unit above normal, vasoactive drug use and AKI-related complications were associated with increased mortality in these patients. Most complications were preventable with appropriate prenatal care.

The incidence of pregnancy-related AKI in the present study was 0.68%, i.e., 68 per 10,000 deliveries. Although PR-AKI is a rare occurrence in high-income countries, its incidence has recently increased [28–31]. For example, the incidence of pregnancy-related AKI in the USA has recently increased from 0.04% in 2006 to 0.12% in 2015, with an overall rate of 0.08% [32]. The rising incidence of pregnancy-related AKI is attributed to higher detection rates, higher hospital delivery rates, an increase in high-risk pregnancies, and higher rates of comorbidities due to advanced maternal age [33]. The overall rate of PR-AKI is decreasing worldwide, indicating an improvement in awareness of antenatal care. The significant improvement in obstetric care and reduction in septic abortions in recent decades may have contributed to the decrease in the burden of this problem worldwide [26]. The incidence of PR-AKI in our study is lower than reports from low- and middle-income countries (4-26%) and from some high-income countries (1-2.8%) [34–36]. Studies from India have shown that the incidence of AKI in pregnancy has decreased in relation to the total number of AKI cases over the last 30 years [37, 38]. Out of 4741 pregnant women, PR-AKI was detected in 132 (2.78%) during the third trimester and postpartum period [25]. The main reasons for the persistently high incidence of PR-AKI in developing countries are septic abortions, overall poor postnatal care, lack of medical facilities in rural areas, and relatively late referral of patients with these conditions. The lower incidence of PR-AKI reflected in our studies may be due to a lack of detection measures, limited available screening tests, and a poor understanding of the disease in low-income countries [39].

The most common causes of PR-AKI in the present study were hypertensive disorders of pregnancy, sepsis and pre-renal AKI due to dehydration and hemorrhage. This is consistent with several studies from developed and developing countries where hypertensive disorders of pregnancy, sepsis and hemorrhage accounted for > 50% of cases of PR-AKI [40, 41]. In our study most of the pregnant mothers at diagnosis of pregnancy-related AKI were in their third trimester, 92 (83%) where the above three are recognized as main causes of PR-AKI in the late third trimester. Early detection and treatment of preeclampsia, sepsis and hemorrhage are limited in low-income countries, including Ethiopia. Timely and aggressive management of obstetric hemorrhage and puerperal sepsis is needed to reduce the burden of such treatable and preventable etiologies of PR-AKI in developing countries.

In low-income countries, inadequate perinatal care and inappropriate treatment of pregnancy-related complications are still the main causes of PR-AKI [35, 42]. In the present study, preeclampsia was the leading obstetric comorbidity in patients with pregnancy-related AKI, occurring in more than two-thirds (71.1%), which is consistent with the fact that preeclampsia is the leading cause of severe PR-AKI and typically occurs in the third trimester and postpartum period [43]. In the same study, a higher burden of preeclampsia was found [43]. The effect of preeclampsia on endothelial damage and vasoconstriction contributes to long-term renal dysfunction and persistent impairment of the glomerular filtration rate [44], as seen in our study, in which partial renal recovery was observed in 16% of cases. In the past, AKI was also considered a completely reversible syndrome, but in recent years, several studies have indicated that AKI may increase the risk of developing CKD, i.e., further damage to the kidneys [44, 45].

HEELP syndrome, considered a continuum of preeclampsia, is a serious disorder in pregnancy and contributor of pregnancy-related AKI. It accounted for 26.7% of PR-AKI in our study, which is consistent with the literature accounting for 15–40% of all cases of PR-AKI [46]. As pre-eclampsia was seen in more than two- thirds (71%) of mothers with PR-AKI, the rate of HELLP syndrome is also expected to be higher than non pre-eclamptic pregnant mothers. HELLP syndrome is also associated with increased maternal morbidity and mortality [47].

Acute kidney injury in pregnancy (PR-AKI) deserves special attention as it poses a risk to two lives (mother and fetus). Maternal and fetal outcomes of concern related to pregnancy-related AKI include permanent renal injury, maternal survival, rate of renal recovery, dialysis dependency, rate of live births, stillbirths, and intrauterine death. In our study, AKI during pregnancy was associated with a higher risk of maternal death [44]. The mortality rate among women with PR-AKI in our study was 7.5%, higher than in developed countries, which range from 2.7 to 4.3% [48], but lower than reports from developing countries, which range from 23 to 33% [27, 49]. However, this result is similar to studies from India, where the reported maternal mortality rate was 6% [50]. The results of this study show that PR- AKI remains an alarming source of morbidity in low-income countries. This calls for a concerted effort by health workers for early detection and prompt treatment of mothers at high risk of AKI and its associated complications.

The majority of women with AKI did not require renal replacement therapy and were treated conservatively. Hemodialysis was needed in 8.6% of patients, which is comparable to studies from China and Nepal (6% and 8%, respectively) [25, 51] but lower than the Moroccan study (16.2%) [52]. In previous studies, a higher percentage (16% in South Africa and 33.3% in Turkey) of pregnant women with AKI needed dialysis treatment [53, 54]. However, in these studies, a higher serum creatinine value was used to confirm the diagnosis of AKI. This could be a possible explanation for the high mortality rates and high dialysis rates in the earlier studies.

The recovery rate of renal function in women with PR- AKI is satisfactory. In our study, 75.6% and 16% of PR-AKI patients had complete and partial recovery of renal function, respectively. The results of our study are consistent with other research findings, which found complete recovery of renal function in 68-89% [5, 25, 55–57]. Early detection and treatment of PR-AKI should favor good outcomes. However, outcome studies suggest that PR-AKI is associated with residual renal dysfunction [34].

In the present study, fetal survival was found in only almost half (56%) of cases with AKI, while IUFD, stillbirth and early neonatal death (ENND) occurred in 10.7%, 17% and 1.6% of cases, respectively. This high fetal mortality rate is consistent with a study from India and China in which stillbirth and perinatal mortality associated with AKI were reported to be 23.5% and 30%, respectively [47, 58]. A more recent study from a tertiary care hospital in Somaliland also revealed high maternal and fetal mortality rates [9.68%; 95% CI, 22.49–27.51] and [58.6%; 95% CI, 1.45–1.99], respectively [59].

Admission to the ICU was 26.7% in our study and was higher in mothers with composite outcomes (52% vs. 19%). This is consistent with studies from Somaliland on PR-AKI, where the need for ICU admission was 26% [59]. Admission to the ICU increases maternal mortality.

The determinants of mortality in PR-AKI were the use of vasoactive agents, preexisting CKD, AKI-related complications and high serum creatinine levels. This finding is consistent with a study from Brazil [60] in which norepinephrine use, hemodialysis therapy, and KDIGO stage 3 were independently associated with higher maternal mortality in patients with PR-AKI.

## Conclusions

Although acute kidney injury is a rare complication of pregnancy, it is associated with high maternal morbidity and mortality. The most common causes of PR-AKI include pregnancy-induced hypertension, dehydration, hemorrhage and sepsis. The prevalence of complete renal recovery in pregnant patients with acute kidney injury was similar to that in other studies. Timely and aggressive treatment of obstetric hemorrhage and puerperal sepsis is needed to reduce the burden of PR-AKI. In developing countries, antenatal care should be emphasized in public health care to prevent septic abortion-related sepsis and pregnancy-induced hypertension [57].

The provision of health facilities with appropriately trained staff and the implementation of prevention strategies will be of great value in reducing the scale of the problem [7]. Our analysis also serves as a stimulus for future studies to investigate improvements in antenatal care and early referral to nephrologists to prevent AKI and mitigate its associated consequences.

### Limitations of the study

To determine the prevalence of PR- AKI in facilities with inadequate resources, this study examined five years of data. Our study has several limitations. First, key Sociodemographic variables were missing from the patient charts. Demographic variables such as marital status, education level, and occupation were not routinely recorded in the medical charts. Second, neonatal status was not routinely recorded in patient notes during postnatal follow-up. The neonatal mortality recorded in this study mainly reflects neonatal death until the mother is discharged from the hospital. Both neonatal and maternal status is routinely recorded in the discharge letter. Some inherent disadvantages of a retrospective cohort should also be noted, such as missing information when using existing records (information bias) or selection bias, as individuals are selected after the outcome has occurred, so the presence of both conditions (exposure and outcome) at the time of data collection may have influenced our study. Using KDIGO definitions for pregnancy-related AKI is reasonable but has limitations as creatinine naturally decreases during mid-pregnancy and there is actually no real consensus on the definition of AKI in pregnancy. Moreover, creatinine tends to rise transiently after delivery in normal pregnancy. This study did not assess CKD risks among those with episodes of PR-AKI as the three months follow up was not available or complete from the registries. Finally, reporting OR in a cohort study is a limitation as it shows association and not causality.

## Data Availability

The datasets used and/or analysed during the current study available from the corresponding author on reasonable request.

## Abbreviations

ACE I: Angiotensin-converting enzyme inhibitors
ACSH: Ayder Comprehensive Specialized Hospital
AFLP: Acute fatty liver of pregnancy
AKI: Acute kidney injury
ALT: Alanine transaminase
APGAR: Appearance, pulse, grimace, activity, and respiration
APH: Antepartum hemorrhage
AOR: Adjusted odd ratio
AST: Aspartate transaminase
CKD: Chronic kidney disease
CLD: Chronic liver disease
DBP: Diastolic blood pressure
ENND: Early neonatal death
GA: Gestational age
HUS: Hemolytic uremic syndrome
HELLP: Hemolysis Elevated Liver enzymes and Low Platelets
ICU: Intensive Care Unit
IQR: Interquartile range
IRB: Institutional Review Board
IUFD: Intrauterine fetal death
KDIGO: Kidney Disease Improving Global Outcomes
LMICs: Low- and middle-income countries
MMR: Maternal mortality rate
MU: Mekelle University
NSAID: Non-steroidal anti-inflammatory drug
PR AKI: Pregnancy-related acute kidney injury
PPH: Postpartum hemorrhage
PR: Pulse rate
RRT: Renal Replacement Therapy
SBP: Systolic blood pressure
TTP: Thrombotic thrombocytopenic purpura
